# Drak Is Required for Actomyosin Organization During *Drosophila* Cellularization

**DOI:** 10.1534/g3.115.026401

**Published:** 2016-01-25

**Authors:** Ashish B. Chougule, Mary C. Hastert, Jeffrey H. Thomas

**Affiliations:** * Department of Cell Biology and Biochemistry, Texas Tech University Health Sciences Center, Lubbock, Texas 79430; † Department of Biological Sciences, Texas Tech University, Lubbock, Texas 79409

**Keywords:** *Drosophila*, cellularization, Drak, actomyosin, furrow canals, death-associated protein kinase

## Abstract

The generation of force by actomyosin contraction is critical for a variety of cellular and developmental processes. Nonmuscle myosin II is the motor that drives actomyosin contraction, and its activity is largely regulated by phosphorylation of the myosin regulatory light chain. During the formation of the *Drosophila* cellular blastoderm, actomyosin contraction drives constriction of microfilament rings, modified cytokinesis rings. Here, we find that Drak is necessary for most of the phosphorylation of the myosin regulatory light chain during cellularization. We show that Drak is required for organization of myosin II within the microfilament rings. Proper actomyosin contraction of the microfilament rings during cellularization also requires Drak activity. Constitutive activation of myosin regulatory light chain bypasses the requirement for Drak, suggesting that actomyosin organization and contraction are mediated through Drak’s regulation of myosin activity. Drak is also involved in the maintenance of furrow canal structure and lateral plasma membrane integrity during cellularization. Together, our observations suggest that Drak is the primary regulator of actomyosin dynamics during cellularization.

During actomyosin contraction, nonmuscle myosin II slides actin bundles over each other, generating force (Vicente-Manzanares *et al.* 2009). Actomyosin contraction is important in many cellular processes, including cell division, differentiation, apoptosis, cell migration, cell adhesion, microvascular permeability, cell shape change, and tissue morphogenesis (Matsumura 2005; Krendel and Mooseker 2005; Sawyer *et al.* 2010). Phosphorylation of the Serine-19, or the Serine-19 and Threonine-18, residues of the myosin regulatory light chain (MRLC) subunit of myosin II is an important regulatory step in both actomyosin assembly and contraction (Vicente-Manzanares *et al.* 2009). These residues correspond to Serine-21 and Threonine-20 in the *Drosophila* MRLC, Spaghetti squash (Sqh) (Jordan and Karess 1997). A variety of serine/threonine kinases, such as Ca^2+^/calmodulin-dependent Myosin Light Chain Kinases (MLCK), Rho kinases (Rok), Citron kinases, and Death-Associated Protein Kinases (DAPK), can phosphorylate MRLC (Matsumura 2005; Vicente-Manzanares *et al.* 2009). However, it is unclear whether these kinases have specific and potentially different roles in the regulation of actomyosin dynamics, and, if so, what these roles are.

Actomyosin dynamics play an important role during *Drosophila* cellularization, a modified form of cytokinesis that occurs during early embryogenesis (Mazumdar and Mazumdar 2002; Thomas and Wieschaus 2004; Royou *et al.* 2004). After fertilization, the *Drosophila* embryo undergoes 13 cycles of nuclear division without cytokinesis. At the end of the 10th division, many of the nuclei move toward the periphery, forming a syncytial blastoderm, and continue dividing for three more cycles. After the 13th nuclear division, the approximately 6000 blastoderm nuclei become separated into cells by the insertion of membrane between the syncytial nuclei to form the cellular blastoderm (Mazumdar and Mazumdar 2002; Foe and Alberts 1983). The cellularization front is the leading edge of membrane invagination between the nuclei, and consists of infoldings of membrane known as furrow canals. Actin and nonmuscle myosin II are organized into a network of microfilament rings at the cellularization front (Mazumdar and Mazumdar 2002; Young *et al.* 1991; Warn and Robert-Nicoud 1990; Lecuit and Wieschaus 2000). During early cellularization, contractile force generated by microfilament rings aids uniform invagination of furrow canals (Thomas and Wieschaus 2004). During late cellularization, constriction of the microfilament rings partially closes the cell bases in a modified form of cytokinesis (Mazumdar and Mazumdar 2002; Young *et al.* 1991; Warn and Robert-Nicoud 1990; Lecuit and Wieschaus 2000).

Although it is clear that actomyosin dynamics are important for proper cellularization, how actomyosin is regulated during cellularization is not well understood. A few genes, such as *Src64B*, *Btk29A*, and *RhoGEF2*, are known to be required for actomyosin contraction during cellularization (Thomas and Wieschaus 2004; Padash Barmchi *et al.* 2005; Grosshans *et al.* 2005; Strong *et al.* 2011). However, the products of these genes do not directly regulate myosin II, and they do not regulate the assembly or organization of myosin II in the microfilament rings. To address the question of how actomyosin dynamics are regulated during cellularization, an analysis of genes that encode direct regulators of MRLC is needed. The most likely candidates include Rok, and the proteins that contain MLCK-like kinase domains in *Drosophila* (Champagne *et al.* 2000; dos Santos *et al.* 2015). One of these proteins is the serine/threonine kinase Drak. It is the only *Drosophila* homolog of the Death-Associated Protein Kinase (DAPK) (Neubueser and Hipfner 2010). Drak functions synergistically with Rho kinase (Rok) to phosphorylate *Drosophila* Sqh, and to regulate epithelial tissue morphogenesis and ommatidia morphogenesis during post-embryonic development (Neubueser and Hipfner 2010; Robertson *et al.* 2012); however, neither study found a role for Drak independent of Rok.

Here, we analyzed the role of Drak in actomyosin regulation during cellularization, and found that Drak plays a role in the organization and function of microfilament rings through MRLC phosphorylation. We also found that Drak plays a role in the maintenance of furrow canal structure during cellularization. To our knowledge, this is the first study to show the function of a DAPK family member independently regulating actomyosin dynamics *in vivo*.

## Materials and Methods

### Fly strains

OreR was used as the wild-type strain. *drak^del^* and *drak^KO^* are loss-of-function alleles that delete the entire coding region of *drak*, and the kinase domain of *drak*, respectively (Neubueser and Hipfner 2010). *sqh^A21^*, *sqh^A20A21^*, *sqh^E21^*, and *sqh^E20E21^* are phosphomimetic transgenes, and nonphosphorylatable transgenes, expressed under the control of the *sqh* promoter (Winter *et al.* 2001; Jordan and Karess 1997).

### Zipper antibody production

A fusion protein of Zipper (Zip, nonmuscle myosin II heavy chain) amino acids 959–1361, and glutathione S-transferase (a gift from A. Sokac), was expressed in *Escherichia coli* DH5α cells from a pGEX-Zip-6P1 plasmid, and purified using glutathione sepharose 4B beads (Amersham Bioscience) (Frangioni and Neel 1993). Two rabbits were injected using standard methods (Panigen, Blanchardville, Wisconsin). IgG were purified by Melon Gel IgG Spin Purification (ThermoFisher), and tested for specificity by Western blotting (1:50,000) and immunofluorescence (1:200).

### Immunofluorescence and image analysis

Embryos were methanol-heat-fixed (Miller *et al.* 1989) and stained with rabbit anti-Zip (1:200), rabbit anti-Anillin (1:100) (Goldbach *et al.* 2010), mouse anti-Nrt (1:10, Developmental Studies Hybridoma Bank, DSHB), and mouse anti-Arm-N27A1 (1:50, DSHB). Embryos were fixed in 4% formaldehyde/phosphate buffer with heptane (Karr and Alberts 1986), and stained with mouse anti-Dlg (1:20; DSHB), and mouse anti-Pnut (1:10; DSHB). Goat anti-mouse and anti-rabbit secondary antibodies were conjugated to Alexa Fluor 488, 546, or 680 (Invitrogen). Embryos were fixed in 8% formaldehyde/phosphate buffer with heptane (Warn and Magrath 1983), and stained with Alexa Fluor 488-conjugated phalloidin (Invitrogen) to visualize F-actin. Embryos were mounted in Aquapolymount (Polysciences, Warrington, Pennsylvania), and imaged using an Olympus FluoView 300 or a Nikon Ti-E A1 confocal microscope. Image analyses were performed using ImageJ (Schneider *et al.* 2012). Circularity index (c = 4πA/p^2^, where A = area, p = perimeter) was determined (Thomas and Wieschaus 2004), and tested using a two-sided Mann-Whitney test. Circularity index data from wild type and *drak^del^* were compared to circularity index data from *drak^del^* lines carrying phosphomimetic *sqh* transgenes, and nonphosphorylatable *sqh* transgenes, using a Kruskal-Wallis test with Dunn’s post-test.

### Western blotting

Embryos were collected in phosphate-buffered saline/0.1% Tween20 (pH 7.4). Cellularizing embryos were selected and crushed in Laemmli Buffer (Bio-Rad) containing 5% beta-mercaptoethanol. Western blots were probed with anti-Sqh1P (1:1000), anti-Sqh2P (1:5000) and anti-Sqh (1:5000) (Wang and Ward 2010; Zhang and Ward 2011), and anti-β-tubulin (1:500). HRP-conjugated goat anti-guinea pig, anti-rat (1:5000; Sigma-Aldrich), and anti-mouse (1:50,000) secondary antibodies (ThemoFisher) were used. Bands were quantified using Image Studio Lite 4.0, and compared using two-way ANOVA with a Tukey post-test.

### Transmission electron microscopy

Embryos were dechorionated with bleach and fixed using the n-heptane permeabilization method (McDonald *et al.* 2000). Embryos were hand-divitellinized, embedded in epon molds, thick-sectioned (1 μm), and stained with 1% methylene blue-azure II to determine developmental stage. Cellularization stage embryos were thin-sectioned (60–90 nm), stained with 4% uranyl acetate and Reynold’s lead citrate, and examined using a Hitachi 8100 STEM electron microscope with an AMT digital camera.

### Data availability

All strains and reagents are available upon request.

## Results

### Drak is required for organization of the actomyosin cytoskeleton during cellularization

**Figure 1:**
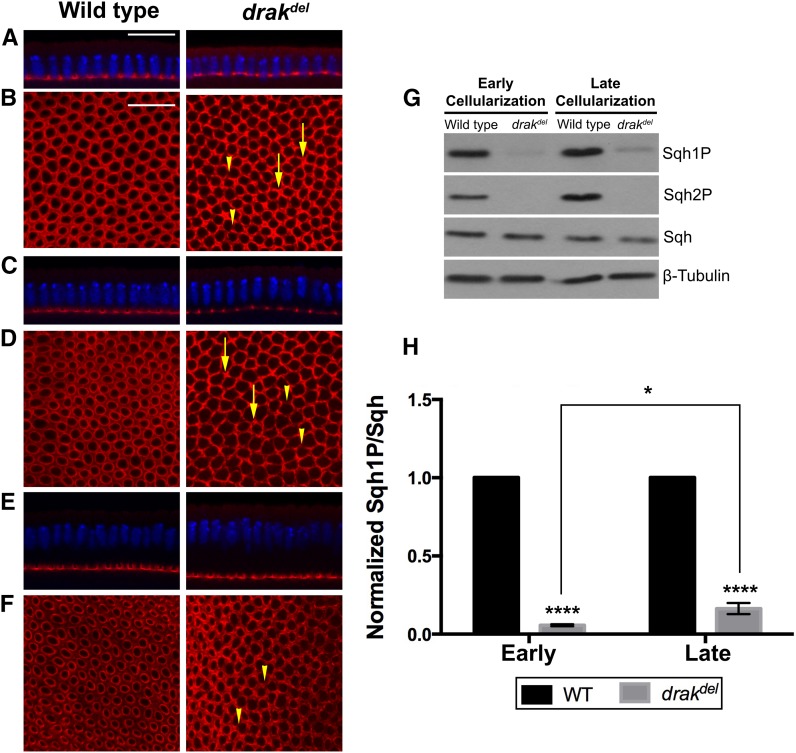
Drak is required for myosin II organization during cellularization. Embryos stained with anti-Zip antibody (myosin II heavy chain, red), and Hoechst (blue), shown in cross-sections (A, C, E), or grazing section projections (B, D, F). (A) Early cellularization front. The depth of the cellularization front varies in *drak^del^* mutant embryos, producing a wavy appearance. (B) Early cellularization microfilament rings. Myosin II is distributed in clumps (arrows), and is missing in some areas (arrowheads) in *drak^del^* mutant embryos. (C) Late cellularization-I front. The cellularization front is wavy in *drak^del^* mutant embryos. (D) Late cellularization-I microfilament rings. *drak^del^* mutant embryos have clumps of myosin II (arrows), and areas lacking myosin II (arrowheads). (E) Late cellularization-II front. The cellularization front is uniform in *drak^del^* mutant embryos, similar to wild type. (F) Late cellularization-II microfilament rings. *drak^del^* mutant embryos have areas lacking myosin II (arrowheads). (G) Western blot showing mono-phosphorylated Sqh (Sqh1P), di-phosphorylated Sqh (Sqh2P), and total Sqh. (H) Plot of relative Sqh1P levels in wild-type embryos and *drak^del^* mutant embryos during early cellularization and late cellularization. Mean ± SD, * *P* < 0.05, **** *P* < 0.0001. Data were compared using two-way ANOVA with a Tukey post-test. Scale bars, 20 μm.

To investigate the function of Drak in actomyosin regulation, we examined *drak* mutants for defects in the actomyosin network that spans the entire embryo during cellularization. We analyzed *drak^del^*, a complete deletion of *drak*, and *drak^KO^*, a deletion of the kinase domain of Drak. Homozygous *drak^del^* and *drak^KO^* mutants are viable and fertile (Neubueser and Hipfner 2010). During early cellularization, before the cellularization front has passed the nuclei, maternally and zygotically homozygous *drak^del^* and *drak^KO^* mutant embryos showed a slightly wavy cellularization front, where furrow canals have different depths (Figure 1A and Supporting Information, Figure S1A). This defect is similar to the irregular early cellularization front defect in *src64* mutant embryos (Thomas and Wieschaus 2004; Strong *et al.* 2011). Myosin II was distributed evenly in the microfilament rings at the cellularization front in wild-type embryos, whereas myosin II was distributed in clumps interspersed between regions of reduced or no myosin II in the microfilament ring network in both *drak^del^* and *drak^KO^* mutant embryos (Figure 1B and Figure S1B). This defect is strikingly different from the microfilament ring defect of *src64* mutant embryos, in which myosin II is distributed evenly throughout the microfilament rings, even though contraction is defective and microfilament ring shapes are abnormal (Strong *et al.* 2011; Thomas and Wieschaus 2004; Strong and Thomas 2011). These results suggest that Drak is involved in the organization, assembly or maintenance of myosin II in microfilament rings during cellularization.

Since *drak^del^* and *drak^KO^* caused similar defects, we conducted further phenotypic analyses with *drak^del^*. During late cellularization when the cellularization front has passed the nuclei, and the rings are undergoing basal closure, wild-type embryos had an even distribution of myosin II in microfilament rings. During late cellularization, *drak^del^* mutant embryos had myosin II organization defects, where myosin II was distributed unevenly in clumps with regions of reduced myosin II. These defects were similar to, but less severe than, those in early cellularization (Figure 1, C–F). We divided late cellularization into an earlier part, late cellularization-I, when the cellularization front had just passed the nuclei, and a later part, late cellularization-II, when the cellularization front was near its final depth, and microfilament rings were highly constricted in wild type (Figure 1, C–F). During late cellularization-I, myosin II was distributed unevenly throughout the microfilament rings in *drak^del^* mutant embryos, but was absent in fewer regions than during early cellularization. Clumps of myosin II were observed in some regions (Figure 1D). During late cellularization-II, some regions of the microfilament rings were missing myosin II, but these were fewer than during late cellularization-I, and there were fewer clumps of myosin II (Figure 1F). Thus, the myosin II organization defects of *drak^del^* became less severe during later stages of cellularization. These data suggest that Drak is required for organization of myosin into contractile rings.

### Sqh phosphorylation is decreased in drak mutants

Mono-phosphorylation (Serine-21) and di-phosphorylation (Serine-21 and Threonine-20) of Sqh were decreased substantially in *drak* mutants during cellularization (Figure 1G and Figure S1C). Since *drak^del^* mutant embryos showed more severe defects during early cellularization than during late cellularization, we compared the phosphorylation levels of Sqh between early cellularization and late cellularization. Levels of mono-phosphorylated Sqh were reduced by 94% during early cellularization, and by 84% during late cellularization in *drak^del^* mutant embryos relative to wild-type embryos (Figure 1H). These results suggest that Drak regulates Sqh phosphorylation during cellularization, but has a greater role during early cellularization, and that less severe defects in myosin II organization during late cellularization were due to increased phosphorylation levels of Sqh in *drak^del^* mutant embryos. By late cellularization, other kinases that phosphorylate MRLC might act synergistically with Drak. We examined Sqh phosphorylation during other stages of early embryogenesis, and found that mono-phosphorylation of Sqh was decreased substantially in *drak^del^* mutant embryos before cellularization. Sqh mono-phosphorylation was also decreased during gastrulation, but not as severely as during cellularization (Figure S2). Thus, during embryogenesis, Drak is either the major regulator of myosin II activity or its function is required for the organization of myosin II and subsequent activity of other kinases that phosphorylate Sqh during cellularization.

### Anillin localization is not dependent on Drak

**Figure 2:**
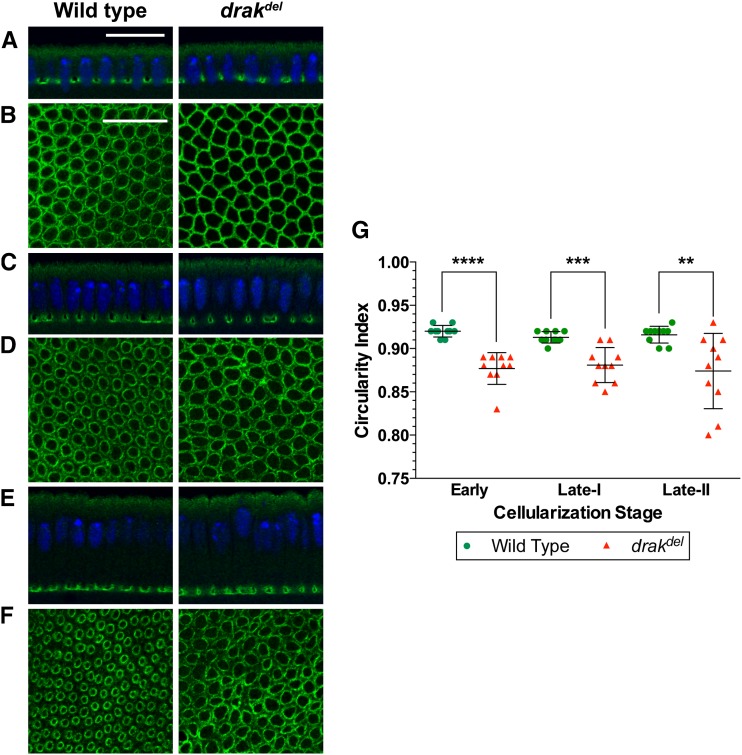
Drak is required for actomyosin contraction during cellularization. Embryos stained with anti-Anillin antibody (green), and Hoechst (blue), shown in cross-sections (A, C, E), or grazing section projections (B, D, F). (A) Early cellularization front. Anillin localization in *drak^del^* mutant embryos is similar to wild-type. (B) Early cellularization microfilament rings. Anillin distribution within the microfilament rings in *drak^del^* mutant embryos is similar to wild-type. Microfilament rings are more angular in *drak^del^* mutant embryos than in wild-type embryos. (C) Late cellularization-I front. Anillin localization in *drak^del^* mutant embryos is similar to wild type. (D) Late cellularization-I microfilament rings. Anillin distribution within the microfilament rings of *drak^del^* mutant embryos is similar to Anillin distribution in wild-type. Microfilament rings are more irregular and less constricted in *drak^del^* mutant embryos than in wild-type embryos. (E) Late cellularization-II front. Anillin localization in *drak^del^* mutant embryos is similar to wild-type. (F) Late cellularization-II microfilament rings. Anillin distribution within the microfilament rings of *drak^del^* mutant embryos is similar to that in wild-type embryos. Microfilament rings are more irregular in *drak^del^* mutant embryos than in wild-type embryos. Microfilament rings are highly constricted in wild-type embryos but show less constriction in *drak^del^* mutant embryos. (G) Plot of microfilament ring circularity indices. Microfilament rings deviate more from circularity in *drak^del^* mutant embryos than in wild-type embryos. Mean ± SD, ** *P* < 0.01, *** *P* < 0.001, **** *P* < 0.0001. Data were compared using a two-sided Mann-Whitney test. Scale bars, 20 μm.

Anillin is a scaffolding protein that binds septins, F-actin, and myosin II, is localized to the furrow canals, and is involved in F-actin and myosin II organization during cellularization (Field *et al.* 2005; Field and Alberts 1995; Thomas and Wieschaus 2004). To determine whether the defective organization of myosin II in *drak^del^* mutant embryos is caused by defects in the localization or organization of Anillin at the cellularization front, we stained the microfilament rings in *drak^del^* mutant embryos with anti-Anillin antibody. We found that Anillin was localized normally to the furrow canals throughout cellularization (Figure 2, A, C, and E). Anillin was distributed uniformly and organized properly in the microfilament rings of *drak^del^* mutant embryos during early cellularization (Figure 2B). During late cellularization, Anillin distribution within the microfilament rings of *drak^del^* mutant embryos was similar to Anillin distribution in wild-type embryos (Figure 2, D and F). Therefore, Anillin localization to the cellularization front, and Anillin distribution within the microfilament rings, do not depend on Drak activity.

### Actomyosin contraction requires Drak

Because, unlike myosin II, Anillin was not disorganized in *drak^del^* mutant embryos, we were able to use it as a marker for microfilament ring shape to test for defects in microfilament ring contraction. Microfilament rings in *drak^del^* mutant embryos had angular and irregular shapes during early cellularization, and had irregular shapes and showed reduced constriction during late cellularization, suggesting an actomyosin contraction defect (Figure 2, B, D, and F). To quantify the ring shape defects, we compared circularity indices of *drak^del^* microfilament rings to those of wild-type microfilament rings (Thomas and Wieschaus 2004; Strong *et al.* 2011). *drak^del^* mutant embryos showed lower circularity indices than wild-type embryos throughout cellularization (Figure 2G). The deviation from circularity compared to wild-type indicated that contractile activity of the microfilament rings was impaired by *drak* mutation. However, observation of some microfilament ring constriction indicated that actomyosin contraction was not completely abrogated by loss of *drak* function. These results suggest that Drak is necessary for actomyosin contraction during cellularization.

### Activated Sqh rescues the drak^del^ phenotype

**Figure 3:**
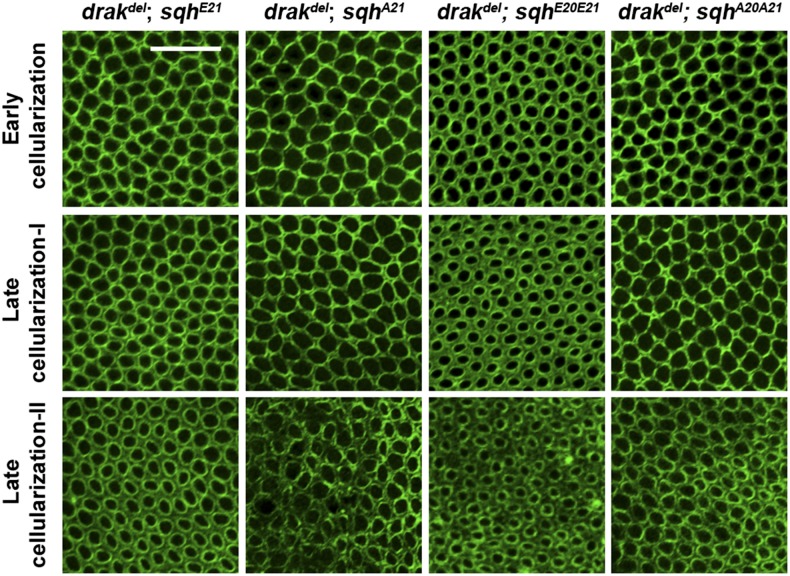
Drak regulates actomyosin organization through myosin II activation. Anti-Zip antibody-stained *drak^del^* mutant embryos expressing Sqh^E21^, Sqh^E20E21^, Sqh^A21^, or Sqh^A20A21^. Throughout cellularization, myosin II is well organized within the microfilament rings of *drak^del^;sqh^E21^* and *drak^del^;sqh^E20E21^* embryos, but is disorganized within the microfilament rings of *drak^del^;sqh^A21^* and *drak^del^;sqh^A20A21^* embryos.

To test whether MRLC phosphorylation is involved in Drak function, we expressed activated Sqh in *drak^del^* mutant embryos. Expression of either the mono-phosphorylated mimetic Sqh^E21^, or the di-phosphorylated mimetic Sqh^E20E21^, completely rescued defective myosin II organization during cellularization, whereas expression of nonphosphorylatable Sqh^A21^ or Sqh^A20A21^ did not. An even distribution of myosin II throughout the microfilament rings similar to wild-type embryos was observed in *drak^del^* mutant embryos expressing activated Sqh, whereas *drak^del^* mutant embryos expressing nonphosphorylatable Sqh, Sqh^A21^, or Sqh^A20A21^, showed myosin II organization defects similar to *drak^del^* mutant embryos (Figure 3). *drak^del^* mutant embryos expressing activated Sqh also showed uniform cellularization fronts similar to those of wild-type embryos (data not shown). These results suggest that Drak regulates myosin II organization through the phosphorylation of Sqh.

**Figure 4:**
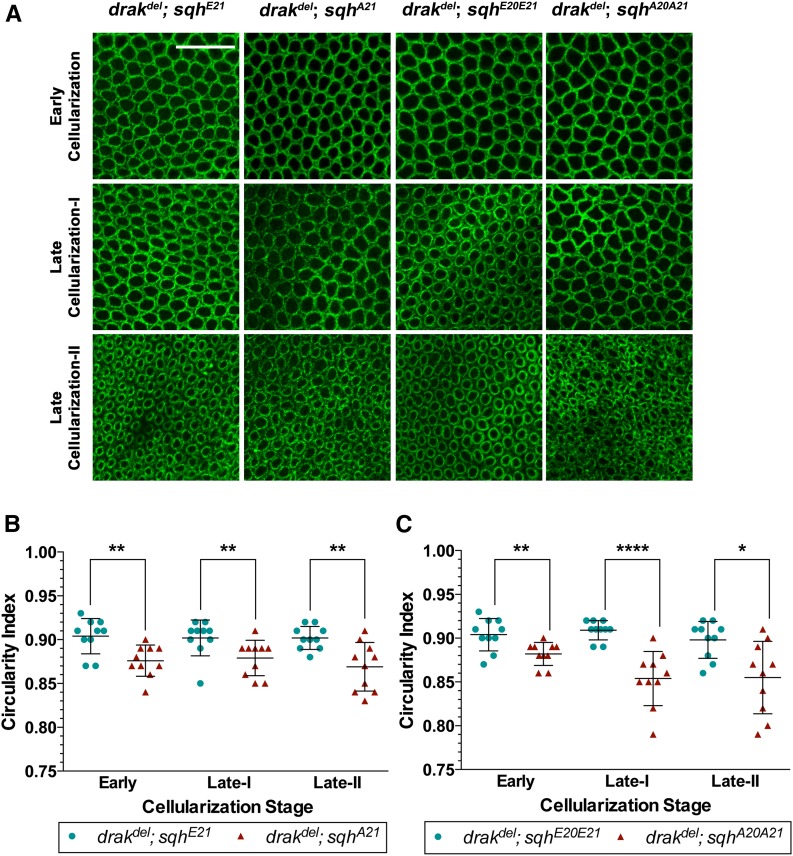
Actomyosin contraction is restored in *drak* mutants by myosin II activation. (A) Anti-Anillin antibody-stained *drak^del^* mutant embryos expressing Sqh^E21^, Sqh^E20E21^, Sqh^A21^, or Sqh^A20A21^. Microfilament rings in *drak^del^;sqh^E21^* and *drak^del^;sqh^E20E21^* embryos are more rounded and regular in shape than microfilament rings in *drak^del^;sqh^A21^* and *drak^del^;sqh^A20A21^* embryos. (B, C) Plots of circularity indices of microfilament rings of anti-Anillin antibody-stained *drak^del^* mutant embryos expressing Sqh^E21^ or Sqh^A21^ (B), and *drak^del^* mutant embryos expressing Sqh^E20E21^ or Sqh^A20A21^ (C). The circularity index is higher in embryos expressing Sqh^E21^ compared to embryos expressing Sqh^A21^ (B), and in embryos expressing Sqh^E20E21^ compared to embryos expressing Sqh^A20A21^ (C). Mean ± SD, * *P* < 0.05, ** *P* < 0.01, **** *P* < 0.0001. Data were compared using a two-sided Mann-Whitney test. Scale bars, 20 μm.

We analyzed microfilament ring contraction in Anillin-stained embryos. Expression of activated Sqh in *drak^del^* mutant embryos restored microfilament ring shape, whereas expression of nonphosphorylatable Sqh did not (Figure 4A). Microfilament ring circularity indices of *drak^del^*;*sqh^E21^* embryos were higher than those of *drak^del^*;*sqh^A21^* embryos throughout cellularization. Likewise, *drak^del^*;*sqh^E20E21^* circularity indices were higher than *drak^del^*;*sqh^A20A21^* circularity indices (Figure 4, B and C). Circularity indices of *drak^del^*;*sqh^E21^* and *drak^del^*;*sqh^E20E21^* embryos did not show any significant difference from those of wild-type embryos (Figure 2G and Figure 4, B and C). We did not observe any enhancement of the *drak^del^* phenotype by expression of Sqh^A21^ or Sqh^A20A21^ when compared to *drak^del^* alone (Figure 2G and Figure 4, B and C). Thus, activation of Sqh restores both the myosin II distribution and organization defects, and the actomyosin contraction defects. Together, these data suggest that Drak regulates both myosin II organization in microfilament rings, and actomyosin contraction by phosphorylating Sqh.

### Drak is required for furrow canal structure and membrane integrity

**Figure 5:**
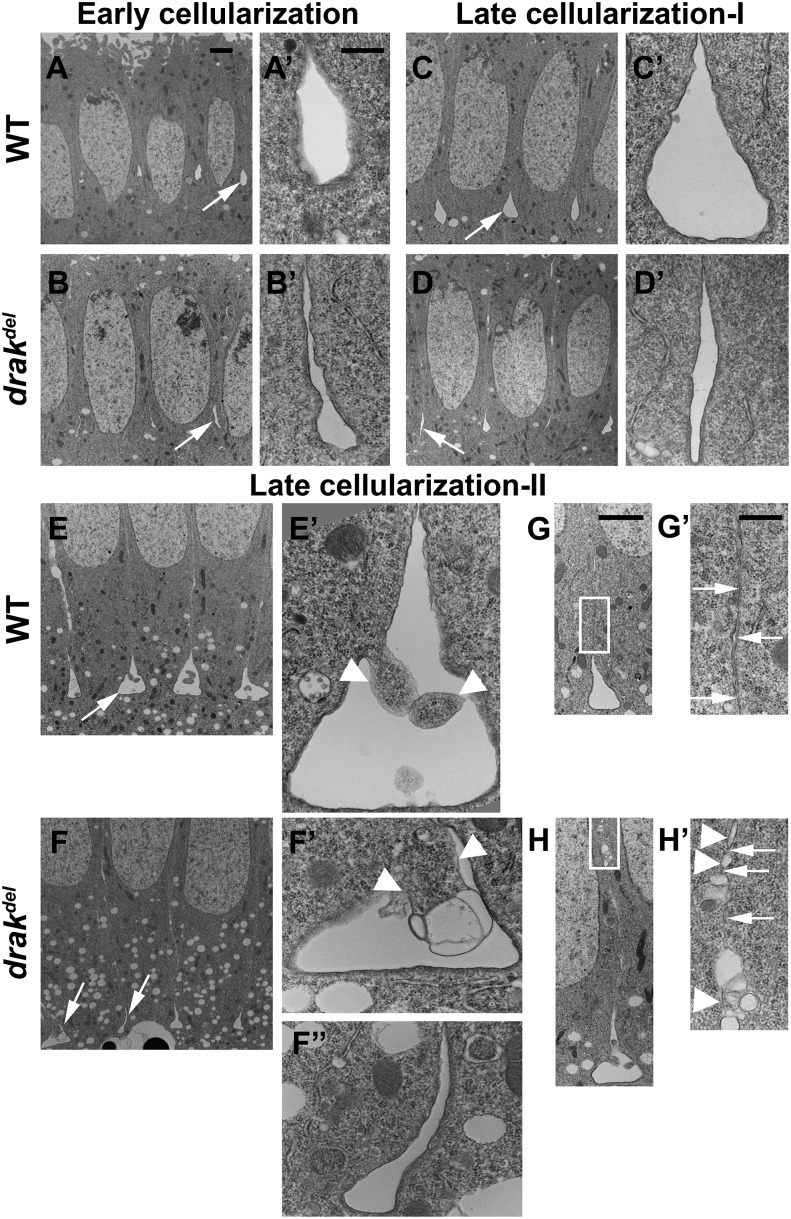
Drak is required for furrow canal structure and plasma membrane integrity. Transmission electron micrographs. (A, B) Early cellularization. Wild-type furrow canals are teardrop-shaped (arrow in A, A’), *drak^del^* furrow canals are narrow (arrow in B, B’). (C, D) Late cellularization-I. Wild-type furrow canals are teardrop-shaped (arrow in C, C’), *drak^del^* furrow canals are narrow (arrow in D, D’). (E, F) Late cellularization-II. Wild-type furrow canals are expanded with broad, flat bases (arrow in E, E’). Blebs are present in wild-type embryos (arrows in E’). *drak^del^* furrow canals are either expanded with membrane blebbing (arrow in F, F’), or narrow (arrow in F, F”). Blebs are larger in *drak^del^* mutant embryos (arrowheads in F’). (G) In late cellularization-II wild-type embryos, plasma membranes are closely apposed (arrowheads, G’). (H) In late cellularization-II *drak^del^* mutant embryos, some membrane regions consist of strings of vesicles (arrowheads, H’), with cytoplasmic connections between the cells (arrows, H’). Scale bars, 2 μm (A–H), 500 nm (A’–H’, F”).

Since *drak^del^* mutant embryos showed defects in the organization of the cytoskeleton in the microfilament rings, we thought that furrow canal structure might be abnormal in *drak* mutant embryos. Therefore, we analyzed the furrow canal structure in more detail using transmission electron microscopy. During early cellularization, wild-type embryos had teardrop-shaped furrow canals. However, most furrow canals in *drak^del^* mutant embryos were narrow at the bases, and appeared collapsed. A few furrow canals in *drak^del^* mutant embryos contained blebs (Figure 5, A, A’, B, and B’). Unexpanded furrow canals in *drak^del^* mutant embryos during early cellularization are consistent with defective actomyosin contraction. In the absence of actomyosin contraction in the microfilament network, the furrow canals are not pulled into their normal teardrop shapes.

During late cellularization, wild-type embryos had flask-shaped furrow canals, with broader bases than during early cellularization. A few furrow canals showed membrane blebbing (Figure 5, C, C’, E, and E’). In *drak^del^* mutant embryos, some furrow canals were unexpanded, whereas many were broad and flat-bottomed like wild type (Figure 5, D, D’, F, F’, and F’’). During late cellularization-I, *drak^del^* mutant embryos showed some blebbing, similar to wild-type embryos (data not shown). However, during late cellularization-II, some furrow canals in *drak^del^* mutant embryos showed more severe membrane blebbing and larger blebs than wild-type embryos (Figure 5, F and F’). The failure of many furrow canals to expand in *drak^del^* mutant embryos during late cellularization is consistent with the absence of actomyosin ring constriction, which is necessary to pull the furrow canals into flask-like shapes as the basal sides of the cells start to close. Membrane blebbing inside the furrow canals is consistent with a loss of furrow canal membrane integrity, or a loss of cortical cytoskeleton integrity in *drak^del^* mutant embryos.

**Figure 6:**
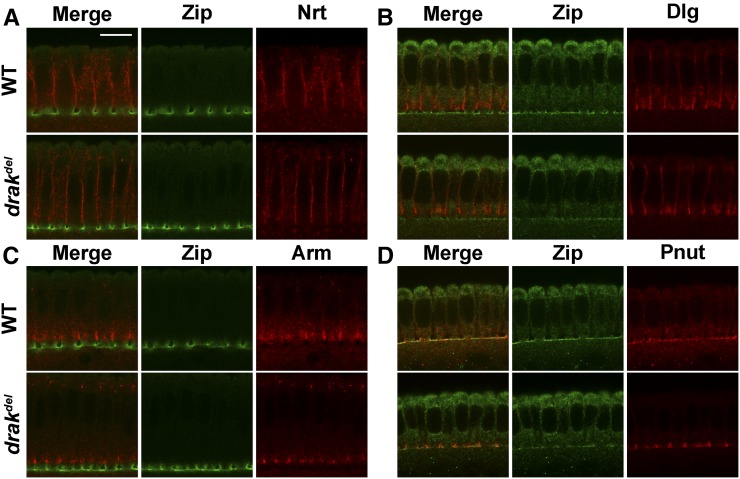
*drak^del^* mutant embryos do not show defects in the localization of membrane proteins. Embryos were stained with anti-Zip (green) (A–D), anti-Nrt (A), anti-Dlg (B), anti-Arm (C), and anti-Pnut (D) (red) antibodies and Hoechst (blue) (A–H). Cross-sections of wild-type embryos and *drak^del^* mutant embryos are shown. (A–C) *drak^del^* mutant embryos show normal localization of Neurotactin (A), Discs large (B), and Armadillo (C) at newly formed lateral plasma membrane apical to the furrow canals, similar to wild-type embryos (A–C, respectively). (D) *drak^del^* mutant embryos show normal localization of Peanut at furrow canals similar to wild-type embryos. Anti-Zip does not stain the furrow canals in formaldehyde-fixed embryos (B and D) as well as it does in heat-methanol fixed embryos (A and C). Scale bar, 20 μm.

Wild-type embryos had closely apposed lateral plasma membranes apical to the furrow canals (Figure 5, A, C, E, G, and G’). We observed that, during late cellularization-II, *drak^del^* mutant embryos had strings of vesicles instead of lateral plasma membranes in many regions, generating cytoplasmic connections between cells (Figure 5, F, H, and H’). Vesiculated plasma membrane and furrow canal defects could be caused by abnormal membrane compartmentalization. We tested this by assaying the localization of proteins associated with different cell membrane compartments during cellularization: Neurotactin (Nrt), Discs large (Dlg), Armadillo (Arm), Anillin (Ani), and Peanut (Pnut) (Sokac and Wieschaus 2008b; Field *et al.* 2005; Lecuit and Wieschaus 2000). Localization of these proteins in *drak^del^* mutant embryos was similar to that of wild-type embryos during late cellularization (Figure 2E and Figure 6). These observations suggest that Drak is essential for maintenance of lateral plasma membrane integrity during cellularization, but not for membrane compartmentalization.

### Actin is distributed within the actomyosin rings independently of Drak activity

**Figure 7:**
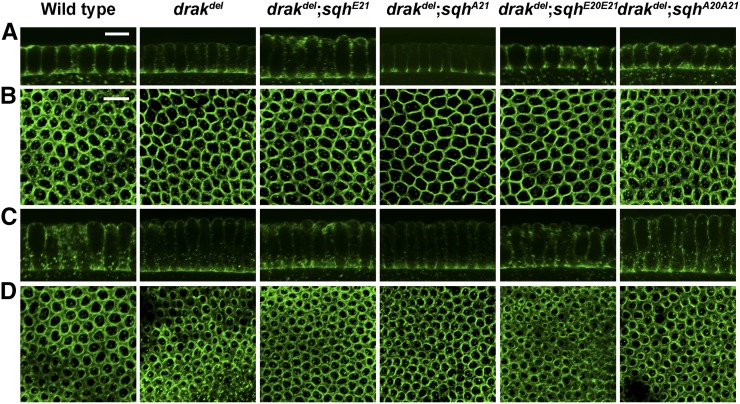
F-actin distribution. Wild-type, *drak^del^*, and *drak^del^* embryos expressing Sqh^E21^, Sqh^E20E21^, Sqh^A21^, or Sqh^A20A21^ stained with phalloidin in cross-sections (A, C), and grazing section projections (B, D). (A) Early cellularization front. F-actin is localized normally to the furrow canals of all genotypes. (B) F-actin distribution in microfilament rings during early cellularization. F-actin is distributed uniformly in the microfilament rings of all genotypes. (C) Late cellularization front. F-actin is localized normally to the furrow canals of all genotypes. (D) F-actin distribution in microfilament rings during late cellularization. Wild-type embryos and *drak^del^;sqh^E21^* embryos show uniform distribution of F-actin in the microfilament rings. F-actin is not distributed evenly within the microfilament rings of *drak^del^*, *drak^del^*;*sqh^A21^*, *drak^del^;sqh^E20E21^*, and *drak^del^;sqh^A20A21^* embryos. Scale bar, 10 μm.

Since myosin II is highly disorganized, and furrow canal structure is defective in *drak^del^* mutant embryos, we wondered whether F-actin was also disorganized in *drak* mutant embryos. To determine whether Drak activity was required for F-actin organization, we examined the distribution of F-actin in microfilament rings. In wild-type embryos and *drak^del^* mutant embryos, F-actin was highly enriched at the furrow canals (Figure 7, A and C). Thus, F-actin is localized normally to the cellularization front in the absence of Drak activity. F-actin was distributed uniformly in microfilament rings in both wild-type embryos and *drak^del^* mutant embryos during early cellularization (Figure 7B). During late cellularization, F-actin was not distributed evenly within the microfilament rings in *drak^del^* mutant embryos (Figure 7D). The F-actin distribution defect is not as severe as the myosin II distribution defect (Figure 1, D and F). Interestingly, F-actin distribution defects were not observed during early cellularization when myosin II organization defects were strongest, but were present during late cellularization when myosin II organization defects were somewhat less pronounced in *drak* mutant embryos. Since F-actin was not found in clumps and gaps like myosin II, we conclude that neither Drak nor myosin II organizes F-actin within the microfilament rings. We speculate that the F-actin distribution defects are a consequence of the furrow canal defects in *drak^del^* mutant embryos.

We tested whether the F-actin distribution defect in the microfilament rings was caused by the absence of Drak-mediated Sqh phosphorylation by expressing phosphomimetic Sqh in *drak^del^* mutant embryos. Expression of Sqh^E21^ rescued the *drak^del^* F-actin distribution defect, but expression of Sqh^A21^ did not rescue the *drak^del^* F-actin distribution defect during late cellularization (Figure 7D). Expression of Sqh^E20E21^ did not completely rescue the *drak^del^* F-actin distribution defects during late cellularization (Figure 7D). This is surprising, in that expression of Sqh^E20E21^ did rescue both the myosin II organization defects, and the actomyosin contraction defects of *drak^del^* mutant embryos (Figure 2, D and F, and Figure 3).

## Discussion

### Actomyosin organization within the microfilament rings requires drak activity

Tight regulation of actomyosin is likely critical for many cellular processes, but how this is accomplished is as yet poorly understood. A key input to the regulation of myosin II is through phosphorylation of the Serine-19, or the Serine-19 and Threonine-18, residues of MRLC. The variety of MRLC kinases might allow different specific aspects of actomyosin dynamics, such as localization, organization, and contraction to be regulated independently. Such a system would provide greater flexibility and control than either a single kinase, or multiple kinases acting in concert, regulating all of these functions. We find that *drak* is required for the organization of myosin II into contractile rings, but is not required for localization of myosin to the cellularization front. Since the majority of Sqh phosphorylation during cellularization is dependent on *drak* activity, Drak either regulates most aspects of myosin II dynamics during cellularization, or Drak-regulated myosin II organization is required for further function of myosin II, such as contraction.

Myosin II is somewhat less disorganized, and Sqh phosphorylation is slightly increased, during late cellularization in *drak* mutants, suggesting that phosphorylation of myosin II by other kinases occurs during late cellularization. Thus, other kinases might act synergistically with Drak to regulate actomyosin organization during late cellularization. For example, Drak function has been shown to be partially redundant with Rok function during later development (Neubueser and Hipfner 2010; Robertson *et al.* 2012). An alternative possibility is that other kinases that do not normally function in myosin II organization in the microfilament rings might phosphorylate Sqh to some degree, and lead to some organization of myosin II in the absence of Drak activity.

Myosin II has been implicated in actin bundling and F-actin organization in some contexts (Vicente-Manzanares *et al.* 2008, 2011; Anderson *et al.* 2008; Brodu and Casanova 2006; Ma *et al.* 2012; Thoresen *et al.* 2011). Since F-actin appears to be organized normally within *drak* mutant microfilament rings during early cellularization, we conclude that myosin II does not play a role in initially organizing F-actin within the microfilament rings during cellularization. F-actin is somewhat disorganized during late cellularization in *drak^del^* mutant embryos, but not as severely as myosin II, nor does the pattern of F-actin distribution fit the pattern of myosin II distribution in *drak^del^* mutant embryos. These observations suggest that F-actin disorganization is an indirect consequence of Drak regulation of myosin II activity, and that F-actin disorganization might be due to actomyosin contraction defects, or furrow canal structural defects.

Anillin is required for the organization of actomyosin contractile rings during cellularization and cytokinesis (Field and Alberts 1995; Field *et al.* 2005; Straight *et al.* 2005; Thomas and Wieschaus 2004). *scraps* (*scra*, *anillin*) mutant embryos have a myosin II organization defect somewhat similar to that of *drak* mutant embryos: myosin II is found in discrete bars in the actomyosin network (Field *et al.* 2005; Thomas and Wieschaus 2004). Despite this similarity, myosin II defects differ between *scra* and *drak* mutant embryos. Myosin II becomes more disorganized during late cellularization in *scra* mutant embryos (Thomas and Wieschaus 2004). Myosin II becomes slightly better organized during late cellularization in *drak* mutant embryos. This organizational difference is likely caused by actomyosin contraction during microfilament ring constriction occurring in a highly disorganized cytoskeleton in *scra* mutant embryos, and occurring in a disorganized cytoskeleton that has slightly improved during constriction in *drak* mutant embryos. Anillin interacts with myosin II only when MRLC is phosphorylated (Straight *et al.* 2005). Together with our results, this suggests that Drak phosphorylation of Sqh might be necessary for Anillin-mediated myosin II organization within the contractile ring.

### drak is required for actomyosin contraction

Phosphorylation of MRLC on Serine-19, or Serine-19 and Threonine-18, leads to the unfolding of inactive myosin II hexamers into an open conformation that allows assembly of bipolar myosin II filaments and their association with F-actin to form actomyosin filaments (Vicente-Manzanares *et al.* 2009). This is likely how Drak organizes myosin II, as described above. Phosphorylation of MRLC on Serine-19, or Serine-19 and Threonine-18, also leads to the activation of the Mg^2+^-ATPase activity of myosin II that slides actin filaments past each other, causing actomyosin contraction (Vicente-Manzanares *et al.* 2009). Three aspects of the *drak* mutant phenotype support the requirement for Drak in actomyosin contraction: wavy cellularization fronts caused by nonuniform furrow canal depths, abnormal microfilament ring shapes, and failure of microfilament rings to constrict during late cellularization. These are the same defects that suggest an actomyosin contraction defect in *src64* mutant embryos. However, *src64* mutant embryos do not show myosin II organization defects (Strong *et al.* 2011; Thomas and Wieschaus 2004). Because effective actomyosin contraction likely requires properly organized actomyosin filaments within the contractile ring apparatus, it is unclear whether Drak directly regulates actomyosin contraction or whether Drak enables actomyosin contraction only through proper organization of myosin II within the microfilament rings. One possibility is that phosphorylation of Sqh by Drak both organizes actomyosin filaments into a contractile ring apparatus, and directs actomyosin contraction. An alternative possibility is that Drak is directly responsible for organizing actomyosin filaments into a contractile ring by phosphorylating Sqh, but Drak is not directly involved in its contraction, and different kinases that phosphorylate Sqh regulate actomyosin contraction. Thus, Drak could be an early regulator of myosin II activity during cellularization, such that further phosphorylation of Sqh and myosin II-driven contraction is dependent on Drak-mediated organization of myosin II. At some level, the regulation of actomyosin contraction diverges from the regulation of actomyosin filament organization: Src64 is required for contraction, but has no role in myosin II organization (Strong *et al.* 2011; Thomas and Wieschaus 2004).

Rescue of myosin II organization, actomyosin contraction, and F-actin distribution defects in *drak* mutant embryos by the mono-phosphorylated Sqh^E21^ phosphomimetic suggests that Drak-mediated mono-phosphorylation of Sqh at Serine-21 is sufficient for regulation of actomyosin dynamics during cellularization. Although the diphosphorylated Sqh^E20E21^ phosphomimetic also rescues myosin II organization, and actomyosin contraction defects, it does not rescue F-actin distribution defects in *drak* mutant embryos. These results are consistent with Drak primarily phosphorylating Sqh at Serine-21, and are consistent with reports that DAPK family members phosphorylate MRLC mainly at Serine-19 (Jin *et al.* 2001; Kuo *et al.* 2003).

### Drak and membrane and cortex integrity

The normal teardrop shape of the furrow canals in early cellularization is likely caused by actomyosin contraction in the microfilament rings (Thomas and Wieschaus 2004). In *drak* mutant embryos, unexpanded early cellularization furrow canals, and the failure of many late cellularization furrow canals to expand further, suggest that Drak is required for proper furrow canal structure. Some of the furrow canal structural defects in *drak* mutant embryos are similar to those of *nullo* mutant embryos: collapsed furrow canals and blebbing (Sokac and Wieschaus 2008b; Postner and Wieschaus 1994). However, *nullo* mutant embryos, as well as *RhoGEF2* or *dia* mutant embryos, have other, more severe, furrow canal defects: missing or regressing furrow canals, and compromised lateral membrane-furrow canal compartment boundaries. Furthermore, cytochalasin treatment causes similar defects, suggesting that reduced F-actin levels in the furrow canals are responsible for these defects (Sokac and Wieschaus 2008a, 2008b; Padash Barmchi *et al.* 2005). Thus Nullo, RhoGEF2 and Dia regulate F-actin and its levels in furrow canals. Our observations suggest that Drak regulates myosin II, and thereby regulates actomyosin organization and contraction, and that these are necessary for structural integrity and expansion of the furrow canals, but not for their continued existence.

The furrow canals of *drak* mutant embryos during late cellularization show extensive blebbing into the lumens. This is consistent with a defect in furrow canal membrane or cortex integrity. Blebs can be formed by local rupture of the cortical cytoskeleton, or detachment of the plasma membrane from the cortical actomyosin cytoskeleton (Charras and Paluch 2008; Cunningham 1995; Keller and Eggli 1998; Paluch *et al.* 2005). Actomyosin contraction has been implicated in bleb formation (Charras and Paluch 2008; Fishkind *et al.* 1991; Hickson *et al.* 2006; Paluch *et al.* 2005, 2006). Therefore, we propose that blebbing in furrow canals is caused by aberrant localized actomyosin contraction during late cellularization in the disorganized actomyosin cytoskeleton of *drak* mutant embryos. Contraction is presumably driven by phosphorylation of Sqh by kinases other than Drak. Since actomyosin contraction occurs in a disorganized actomyosin cytoskeleton, it does not lead to uniform constriction of the microfilament rings, but instead leads to localized contraction, which produces cytoplasmic blebs. However, other causes of furrow canal defects are possible. Plasma membrane attachment sites might not form or function properly in the disorganized furrow canal cytoskeleton in *drak* mutant embryos. The disorganized cytoskeleton might inhibit vesicle trafficking (Sokac and Wieschaus 2008a; Smythe and Ayscough 2006; Mooren *et al.* 2012). Vesicle trafficking itself might be defective: mammalian DAPKs have been shown to be involved in membrane trafficking, and in phosphorylation of syntaxin A1 (Tian *et al.* 2003; Gozuacik and Kimchi 2004).

Vesiculated lateral plasma membrane in *drak* mutant embryos during late cellularization suggests that the plasma membrane breaks down. Intriguingly, *scra* mutant embryos have lines of vesicles where the closely apposed lateral plasma membranes would have been. However, in *scra* mutant embryos, vesiculation is observed during early cellularization, but to a lesser extent than during late cellularization (Field *et al.* 2005). *drak* mutant embryos do not show lateral plasma membrane vesiculation defects until late cellularization. *drak* mutant defects in both the furrow canal membrane and the lateral plasma membrane might reflect a general defect in membrane integrity. It will be interesting to investigate the potential role of myosin II organization in furrow canal structure and plasma membrane integrity.

## Supplementary Material

Supporting Information
