# Supplementary material for: Drak Is Required for Actomyosin Organization During Drosophila Cellularization
Source: G3 (Bethesda). 2016 Jan 25;6(4):819–28. doi: 10.1534/g3.115.026401 (PMC4825652; doi:10.1534/g3.115.026401)
Supplement: Supporting Information [file supp_6_4_819__index.html]

Drak Is Required for Actomyosin Organization During Drosophila Cellularization — Drak Is Required for Actomyosin Organization During Drosophila Cellularization — Supporting Information 

# Drak Is Required for Actomyosin Organization During *Drosophila* Cellularization

## Supporting Information for Chougule, Hastert, and Thomas, 2016

**Files in this Data Supplement:**

- Figure S1 - *drakdel* and *drakKO* defects in myosin II organization during cellularization. (.pdf, 107 KB)
- Figure S2 - Sqh phosphorylation levels in *drakdel* mutant embryos during early embryonic development. (.pdf, 56 KB)
