# Supplementary material for: Drak Is Required for Actomyosin Organization During Drosophila Cellularization
Source: G3 (Bethesda). 2016 Jan 25;6(4):819–28. doi: 10.1534/g3.115.026401 (PMC4825652; doi:10.1534/g3.115.026401)
Supplement: Supporting Information [file supp_g3.115.026401_FigureS1.pdf]

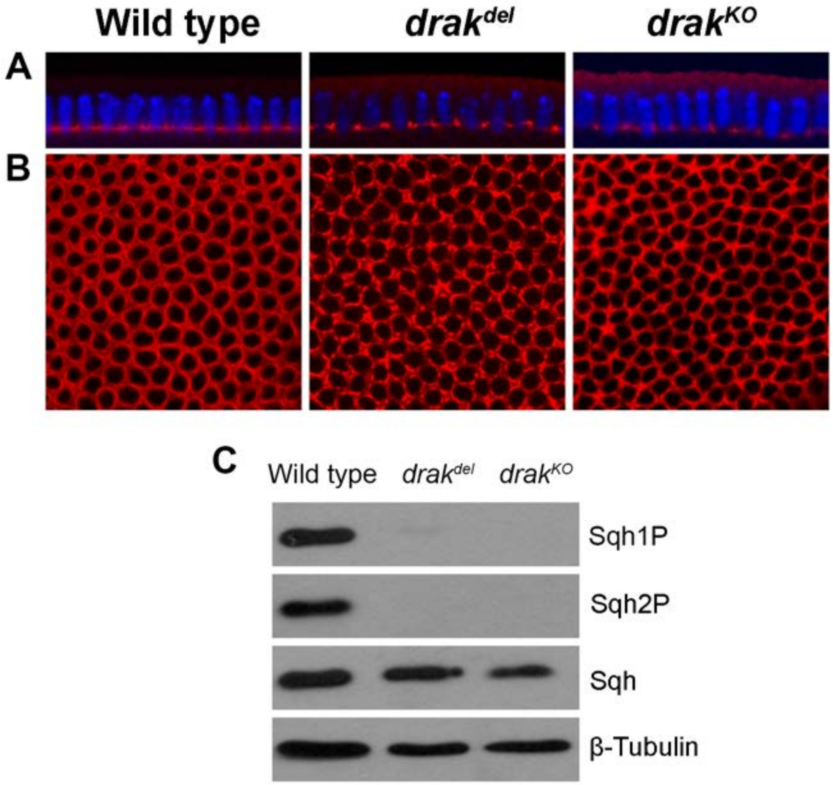

Figure S1 *drak*^{del} and *drak*^{KO} defects in myosin II organization during cellularization. Embryos were stained with anti-Zip antibody (red) and Hoechst (blue). (A) Cross-sections showing the early cellularization front. The cellularization fronts of *drak*^{del} and *drak*^{KO} mutant embryos are wavy because the furrow canals have different depths. (B) Grazing section projections of early cellularizing embryos showing the microfilament network. *drak*^{del} and *drak*^{KO} mutant embryos show clumps of myosin II in some regions of the microfilament rings and absence of myosin II in other regions. (C) Western blot showing decreased mono-phosphorylated (Sqh1P) and di-phosphorylated (Sqh2P) Sqh protein (Sqh) in cellularization-stage *drak*^{del} and *drak*^{KO} mutant embryos. No differences in total Sqh (phosphorylated and non-phosphorylated) levels between *drak*^{del} and *drak*^{KO} mutant embryos, and wild-type embryos were observed. β-Tubulin was used as a loading control. Scale bars, 20 μm.
