# Supplementary material for: Drak Is Required for Actomyosin Organization During Drosophila Cellularization
Source: G3 (Bethesda). 2016 Jan 25;6(4):819–28. doi: 10.1534/g3.115.026401 (PMC4825652; doi:10.1534/g3.115.026401)
Supplement: Supporting Information [file supp_g3.115.026401_FigureS2.pdf]

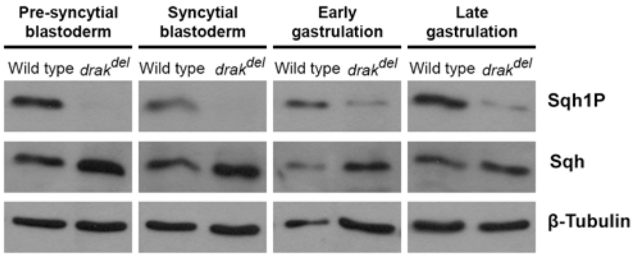

Figure S2 Sqh phosphorylation levels in *drak*^{del} mutant embryos during early embryonic development. Western blot showing strongly decreased mono-phosphorylated Sqh (Sqh1P) in *drak*^{del} mutant embryos compared to wild-type embryos before the syncytial blastoderm stage, and during the syncytial blastoderm, the early gastrulation and the late gastrulation stages. No difference in total Sqh levels between *drak*^{del} mutant and wild-type embryos was observed. β-Tubulin was the loading control.
